# Exosomes derived from mesenchymal stem cells primed with disease-condition-serum improved therapeutic efficacy in a mouse rheumatoid arthritis model via enhanced TGF-β1 production

**DOI:** 10.1186/s13287-023-03523-0

**Published:** 2023-10-04

**Authors:** Eun Wha Choi, I.-Rang Lim, Ji Hong Park, Jiwoo Song, Bongkum Choi, Sungjoo Kim

**Affiliations:** 1 Department of Veterinary Clinical Pathology, College of Veterinary Medicine and Institute of Veterinary Science, Kangwon National University, 1 Kangwondaehak-gil, Chuncheon, Gangwon-do 24341 Republic of Korea; 2 Bioanalysis Center, GenNBio Inc., 700, Daewangpangyo-ro, Bundang-guGyeonggi-do, Seongnam-si, 13488 Republic of Korea; 3 GenNBio Inc., 80, Deurimsandan 2-ro, Cheongbuk-eup, Pyeongtaek-si, Gyeonggi-do 17796 Republic of Korea

**Keywords:** Autoimmune diseases, Collagen-induced arthritis, Exosome, Mesenchymal stem cell, Rheumatoid arthritis

## Abstract

**Backgrounds:**

Rheumatoid arthritis (RA) is a chronic and systemic autoimmune disease characterized by synovial inflammation-mediated progressive destruction of the cartilage and bone, resulting in reduced quality of life. We primed human telomerase reverse transcriptase-overexpressing immortalized human adipose tissue-derived mesenchymal stem cells (iMSCs) with serum derived from a non-human primate RA model and studied the immunomodulatory ability of exosomes obtained from primed iMSCs.

**Methods:**

After immunophenotyping, nanoparticle tracking analysis, and in vitro functional tests, Dulbecco’s phosphate-buffered saline (dPBS, Group C), exosomes derived from the supernatant of iMSCs (Exo-FBS, Group E), exosomes derived from the supernatant of iMSCs primed with RA serum (Exo-RA, Group F), and methotrexate (Group M) were administered in collagen-induced arthritis (CIA) model mice. dPBS was administered to the normal (N) group for comparison (n = 10/group).

**Results:**

Exo-RA had a significantly higher number of exosomes compared to Exo-FBS when measured with nanoparticle tracking analysis or exosome marker CD81, and Transforming growth factor-β1 amounts were significantly higher in Exo-RA than in Exo-FBS. When Exo-FBS or Exo-RA was administered to the collagen-induced arthritis model, serum interleukin (IL)-4 and the proportion of Th2 (CD4+CD25+GATA3+) and M2 (CD11c − CD206+ of CD45+CD64+) cells were significantly increased compared to the control group. Furthermore, Exo-RA could alleviate cartilage damage by significantly lowering the concentrations of proinflammatory cytokines such as tumor necrosis factor-α, keratinocyte chemoattractant, and IL-12p70.

**Conclusion:**

Exosomes derived from disease-condition-serum-primed iMSCs ameliorated cartilage damage in a RA model by enhancing TGF-β1 production, inducing Th2 and M2 polarization and lowering proinflammatory cytokines, TNF-α, KC, and IL-12p70 in the host. Patient-derived serum can be used as an iMSC priming strategy in iMSC-derived exosome treatment of RA.

## Introduction

Rheumatoid arthritis (RA) is a chronic and systemic autoimmune disease characterized by synovial inflammation-mediated progressive destruction of the cartilage and bone, resulting in reduced quality of life. [1]. Currently, methotrexate is the most basic drug for the treatment of rheumatoid arthritis [2]. The side effects of methotrexate are similar to those of other common anticancer drugs [3]. One of the biggest problems in using methotrexate is the development of methotrexate resistance [2]. Several patients do not respond to methotrexate or other antirheumatic drugs (antimalarial drugs, sulfasalazine, and leflunomide). In addition, TNF-α inhibitors, ranked 1st, 2nd, and 3rd in sales in the global biopharmaceutical market, are associated with allergic reactions and reduced efficacy due to anti-drug antibodies [4, 5]. Therefore, it is necessary to develop next-generation treatments that can reduce or replace the existing treatment capacity.

The interactions between mesenchymal stem cells (MSCs) and the host microenvironment regulate the secretion of MSCs. Soluble factors and exosomes from MSCs induce immunomodulatory properties [6]. MSC-based therapy has shown beneficial effects in various animal models of autoimmune diseases and several human clinical trials [7, 8].

Paracrine actions vary depending on the source of the MSCs, target cells, and the microenvironment surrounding the cells [9]. In a previous study, plasma from patients with graft-versus-host disease significantly enhanced the immunosuppressive potential of MSCs. In addition, plasma interferon (IFN)-γ/interleukin (IL)-10 ratio showed a direct correlation with the induction of regulatory T cell (Treg) and immunosuppressive potential [10].

Some risks have been reported with MSC: intra-arterial injection of MSCs has been shown to result in myocardial microinfarction [11], and senescence and loss of stemness of MSCs are major obstacles in clinical application [12, 13].

MSC-derived exosomes (MSC-exosomes) contain various mRNA, miRNAs, and proteins originating from the parent MSCs [14], and display immunoregulatory functions similar to the parent MSCs [15]. MSC-exosomes are 50–150 nm in size [14]; thus, they do not cause micro-infarction problems. MSC-exosomes are stable and easy to store and transport [16]. MSC-exosomes have no risk of immune attack, which is an increasing challenge in MSC transplantation [17, 18].

Telomerase maintains the length of the telomere; hence, it plays an important role in the pluripotency and self-renewal potential of embryonic stem cells [19]. However, the MSCs do not exhibit telomerase activity [20]. The introduction of telomerase into normal human cells induces senescence bypassing and extending the lifespan of cells [21]. Furthermore, the overexpression of human telomerase reverse transcriptase (*hTERT*) in human MSCs increases stemness and inhibits spontaneous differentiation [13].

In the present study, *hTERT*-overexpressing immortalized human adipose tissue-derived MSCs (iMSCs) were used to obtain exosomes. We primed iMSCs with serum derived from a non-human primate RA model and studied the immunomodulatory ability of exosomes obtained from the iMSCs primed with disease-condition-serum.

## Methods

### iMSC culture

iMSCs were purchased from ATCC (SCRC-4000) and maintained in MSC basal medium (PCS-500-030, ATCC) using an MSC growth kit for adipose and umbilical MSCs (PCS-500-040, ATCC) according to the manufacturer’s instructions. De-identified human iMSCs were used in this study with an institutional review board (IRB)-approved exemption (KWNUIRB-2021-04-009-001).

### Immunophenotype characteristics and differentiation capacity of iMSCs

The immunophenotypic characteristics of the iMSCs were analyzed as described in our previous study. iMSCs were seeded at a density of 4.5 × 10^4^ cells/well in a 12-well plate for adipogenesis and osteogenesis. After 4 days, the iMSCs were maintained in adipogenic induction medium (Mesenchymal Adipogenesis Kit, Millipore, Burlington, Massachusetts, USA) or osteogenic induction medium (OsteoDiff Media, StemMACS™, Millipore) for up to 3 weeks, with fresh medium changed every third day. Adipogenesis and osteogenesis were confirmed by staining with Oil Red O (Mesenchymal Adipogenesis Kit; Millipore SCR020) and Alizarin Red S (A5533; Sigma-Aldrich), respectively. For chondrogenesis, 1 × 10^6^ iMSCs were centrifuged in a 15 mL tube to form a pellet, differentiated using cartilage differentiation medium for up to 3 weeks, with fresh media changed every third day (MSC Chondrogenic Differentiation Medium, Promocell, Heidelberg, Germany), fixed in formalin, paraffin-sectioned, and stained with toluidine blue to confirm chondrogenesis.

### Exosome production and characterization

For exosome production, iMSCs were seeded in DMEM supplemented with 2 mmol/mL glutamine, 100 μg/mL penicillin/streptomycin, and 10% fetal bovine serum (FBS) at a density of 10^7^ per 175 T flask (about 6 × 10^4^ cells/cm^2^) for 24 h, and then incubated in DMEM supplemented with 2 mmol/mL glutamine, 100 μg/mL penicillin/streptomycin, and 10% Exosome-depleted FBS (A2720801, Gibco, Grand Island, NY, USA) for 48 h (MSC-FBS). Stored serum samples obtained from non-human primates before the induction of RA (non-RA serum) or during an autopsy after the induction of RA (RA-serum) in a previous non-human primate RA experiment (not yet published; ORIENT-IACUC-21110) were used for priming iMSCs. iMSCs were seeded in DMEM supplemented with 2 mmol/mL glutamine, 100 μg/mL penicillin/streptomycin, 2 mmol/mL glutamine, and 17.5% non-RA or RA serum at a density of 10^7^ per 175 T flask for 24 h, and then incubated in DMEM supplemented with 2 mmol/mL glutamine, 100 μg/mL penicillin/streptomycin, and 10% exosome-depleted FBS for 48 h (MSCs primed with RA serum: MSC-RA).

Exosomes derived from the supernatant of iMSCs (Exo-FBS), iMSCs primed with RA serum (Exo-RA), or non-RA serum (Exo-non-RA) were isolated using the Total Exosome Isolation Reagent (4478359, Invitrogen, Waltham, Massachusetts, USA) according to the manufacturer’s instructions.

The total protein concentration of exosomes was determined by bicinchoninic acid (BCA) assay (BCA protein assay kit™, Pierce), and the size distribution of exosomes was determined by nanoparticle tracking analysis (NTA) using a NanoSight NS300 instrument (Malvern Instruments Ltd., Worcestershire, UK). The morphology of the exosomes was observed using a transmission electron microscopy (TEM) and the expression of CD81 was analyzed using an ExoELISA-ULTRA Complete Kit (EXEL-ULTRA-CD81-1, SBI, Palo Alto, CA, USA).

### ELISA for determination of the concentration of transforming growth factor (TGF)-β1, prostaglandin E2 (PGE2), and IL-1ra in exosomes

The concentrations of TGF-β1, PGE2, and IL-1Ra in exosomes (Exo-FBS and Exo-RA) were assessed using human LAP (TGF-β1) Quantikine ELISA Kit, Prostaglandin E2 Parameter Assay Kit, and Human IL-1ra/IL-1F3 Quantikine ELISA Kit (R&D systems, Minneapolis, MN, USA), respectively.

### Expression levels of MicroRNA (miR)-155-5p, miR-146a-5p, miR-10-5p, miR-142-3p and miR-216a-5p in exosome and iMSCs

miRNAs were isolated from exosomes (Exo-FBS and Exo-RA) and iMSCs using a Total Exosome RNA Protein Isolation Kit (Invitrogen, Waltham, Massachusetts, USA) and Tri-Reagent (MRC), respectively. cDNA was generated using the miRCURY LNA RT kit (Qiagen, Venlo, Netherlands), and qPCR was performed using the miRCURY LNA SYBR Green PCR kit with several miScript primers (Qiagen): hsa-miR-16a-5p, hsa-miR-155-5p, hsa-miR-146a-5p, hsa-miR-10a-5p, hsa-miR-142-3p, and hsa-miR-216a-5p. qPCR was performed according to the protocol provided by the manufacturer. miR-16-5p served as the housekeeping gene and the 2^−ΔΔCT^ method was used to determine expression levels.

### In vitro functional test

#### Experimental animals

Three seven-week-old male DBA/1 mice were obtained from Orient Bio (Gayang, Korea) and acclimatized for one week before the study commenced. This animal study was reviewed and approved by the Institutional Animal Care and Use Committee (IACUC) of Kangwon National University (KW-210824-1).

Chicken type II collagen (CII, Sigma-Aldrich, Burlington, Massachusetts, USA) was used to induce collagen-induced arthritis (CIA). The concentration, preparation, and administration of CII were as described previously [22]. Mice were administered a booster dose three weeks post-first CII administration (Sigma-Aldrich).

### ELISA of multiple cytokines in supernatants of spleen-cell culture obtained from the mice with CIA

Spleen cells from each mouse were isolated on day 48 after CII immunization and cultured as described previously, with or without denatured CII (100 μg/mL), ConA (5 μg/mL), or LPS (2.5 μg/mL) [22]. Each well was treated with the medium (no treatment), Exo-FBS, Exo-RA, or Exo-non-RA. Exosomes and splenocytes were co-cultured at a ratio of 9 μg:2 × 10^5^. This ratio was determined based on a previous study [1]. After culturing for 72 h at 37 °C in a humidified atmosphere, the culture supernatant was collected and stored at -80 °C. Cytokines were measured with the mouse multiplex MAP Kits using the Luminex technology [22].

### In vivo test

#### Experimental animals

Fifty seven-week-old male DBA/1 mice were purchased from Orient Bio (Gayang, Korea) and acclimatized for one week before the commencement of the study. This study was reviewed and approved by the IACUC of GenNBio (GN-IACUC-22-01-10).

#### Experimental groups and induction of CIA

To compare the therapeutic effects of Exo-FBS and Exo-RA in the CIA mouse model, experimental mice were divided into five groups (n = 10 mice per group): normal (N), control (C), Exo-FBS (E), Exo-RA (F), and MTX (M). Mice in the other groups, except the N group, were induced with CIA as described for the in vitro functional test.

#### Treatment protocol

Each mouse in the N and C groups was intravenously administered 150 μL of dPBS, whereas those in the E group were intravenously administered with exosomes derived from the supernatant of 2 × 10^6^ iMSCs (Exo-FBS)/150 μL of dPBS. The F group were intravenously administered with exosomes derived from the supernatant of 2 × 10^6^ iMSCs primed with RA serum (Exo-RA)/150 μL of dPBS twice a week (on days 25, 29, 32, 33, 39, 43, 46, and 50 after CII immunization).

Each mouse in the M group was intraperitoneally administered 3 mg/kg methotrexate (Medac, Hamburg, Germany) three times per week (on days 23, 25, 28, 30, 32, 35, 37, 39, 42, 44, 46, 49, and 51 after CII immunization). All mice were sacrificed on days 52–53 after CII immunization.

### Arthritis severity assessment

The paw thickness of each mouse was measured weekly with a caliper. The arthritis score was evaluated from 0 (no arthritis) to 4 (severe arthritis) thrice per week starting from day 21 post-CII immunization, as described previously [22]: score 0: normal paw; score 1: one or two toes inflamed and swollen; score 2: three or more toes inflamed and swollen; score 3: swelling of the entire paw; score 4: severe swelling of the entire paw and all toes, or ankylosed paw and toes.

### The detection of anti-CII antibody and C-telopeptide of type II collagen (C-telopeptide II) levels

Mice were anesthetized (isoflurane) and sacrificed, and blood samples were collected on day 52 or 53 after CII immunization. The collected sera were stored at − 80 °C until use. Antibodies against chicken CII and mouse CII were measured using a commercial mouse anti-chicken CII ELISA kit (2031, Chondrex Inc., Redmond, WA, USA) and a commercial mouse anti-mouse CII ELISA kit (2036, Chondrex). Serum C-telopeptide II levels were measured in the same way as in the previous paper [23].

### T cell proliferation test using splenocytes from experimental animals

On day 52 or 53, the spleens were harvested from all mice. Splenocytes from each mouse were cultured in a 96-well plate (density: 2 × 10^5^ cells/well and final volume: 200 μL) with or without CII, ConA, or LPS (100 μg/mL, 5 μg/mL, and 2.5 μg/mL respectively). Plates were incubated at 37 °C in 5% CO_2_. After 3 days, a BrdU assay was performed (BrdU labeling time: 6 h) using a commercial kit (Cell Proliferation ELISA BrdU, Colorimetric; Roche Diagnostics, Mannheim, Germany). The stimulation index values (mean optical density of CII- or mitogen-stimulated cultures/mean optical density of medium-only cultures) were calculated for each culture treatment.

### Flow-cytometric determination of T cell subset, CD138 cells, and macrophage subset in the spleen

Splenocytes were obtained from the spleens of DBA/1 mice at autopsy (on day 52 or 53 after CII immunization). An Fc-blocking antibody was used to prevent nonspecific binding (anti-mouse CD16/32; BioLegend, San Diego, CA, USA). Splenocytes were stained with peridinin chlorophyll protein complex-conjugated anti-mouse CD45 (1.25 μL/well, BioLegend), allophycocyanin-conjugated anti-mouse CD3e (1 μL/well, eBioscience, San Diego, CA, USA), fluorescein isothiocyanate conjugated anti-mouse CD4 (2 μL/well, BD Biosciences), and phycoerythrin (PE)-cyanine7-conjugated anti-mouse CD8a (0.5 μL/well, eBioscience). The fraction of CD138-positive cells was analyzed (PE rat anti-mouse CD138, 1 μL/well, BD Biosciences) as described previously [24].

The macrophage subset, the proportion of proinflammatory M1 macrophages (CD45+CD64+CD11c+CD206−) and anti-inflammatory M2 macrophages (CD45+CD64+CD11c−CD206+), was also analyzed as described previously [25].

### Flow-cytometric analysis of the T helper cell subset in the spleen

The proportions of Treg cells (CD4+CD25+Foxp3+), Th17 cells (CD4+CD25+ROR-γt+), Th1 cells (CD4+CD25+T-bet+), and Th2 cells (CD4+CD25+GATA-3+) were analyzed as described previously [22, 24].

### Histopathology

Knee joint tissue sections were stained and cartilage damage was evaluated as described previously [23], and the severity of cartilage damage was graded from 0 (none) to 4 (severe). The knee joints were harvested for histopathological examination after fixation in 10% neutral-buffered formalin, decalcified, and embedded in paraffin.

### Various cytokines in sera and knee joint extracts

Serum and joint extract samples from all mice were used for the analysis of TNF-α, IFN-γ, IL-1α, IL-1β, IL-2, IL-4, IL-6, IL-10, IL-12p70, IL-17, KC, MCP-1, MIP-2, and RANTES with multiplex^®^ MAP Kits (Millipore, Bedford, MA, USA).

Briefly, 12.5 μL of dPBS containing protease inhibitor was added per 1 mg of knee joint sample and homogenized using an automated freeze-crushing cell homogenizer as described in our previous study [23]. Total protein concentrations in the joint extracts were determined using a BCA protein assay kit (Pierce). The total protein concentration of knee joint extracts was adjusted to 2 mg/dL and used for analysis.

### Statistical analysis

All results are expressed as mean ± the standard error of the mean (SEM). The results of the four groups, except for the cytokine data, were compared using one-way analysis of variance (ANOVA), followed by Tukey’s multiple comparison post-hoc tests. Cytokine data were analyzed using Kruskal–Wallis and Dunn’s tests. To compare the means of two related samples, Paired *t*-test was used. *p* < 0.05 was considered significant. All statistical analyses were performed using SPSS version 26.0 (SPSS, Armonk, NY, USA).

## Results

### iMSC immunophenotyping and differentiation

**Fig. 1:**
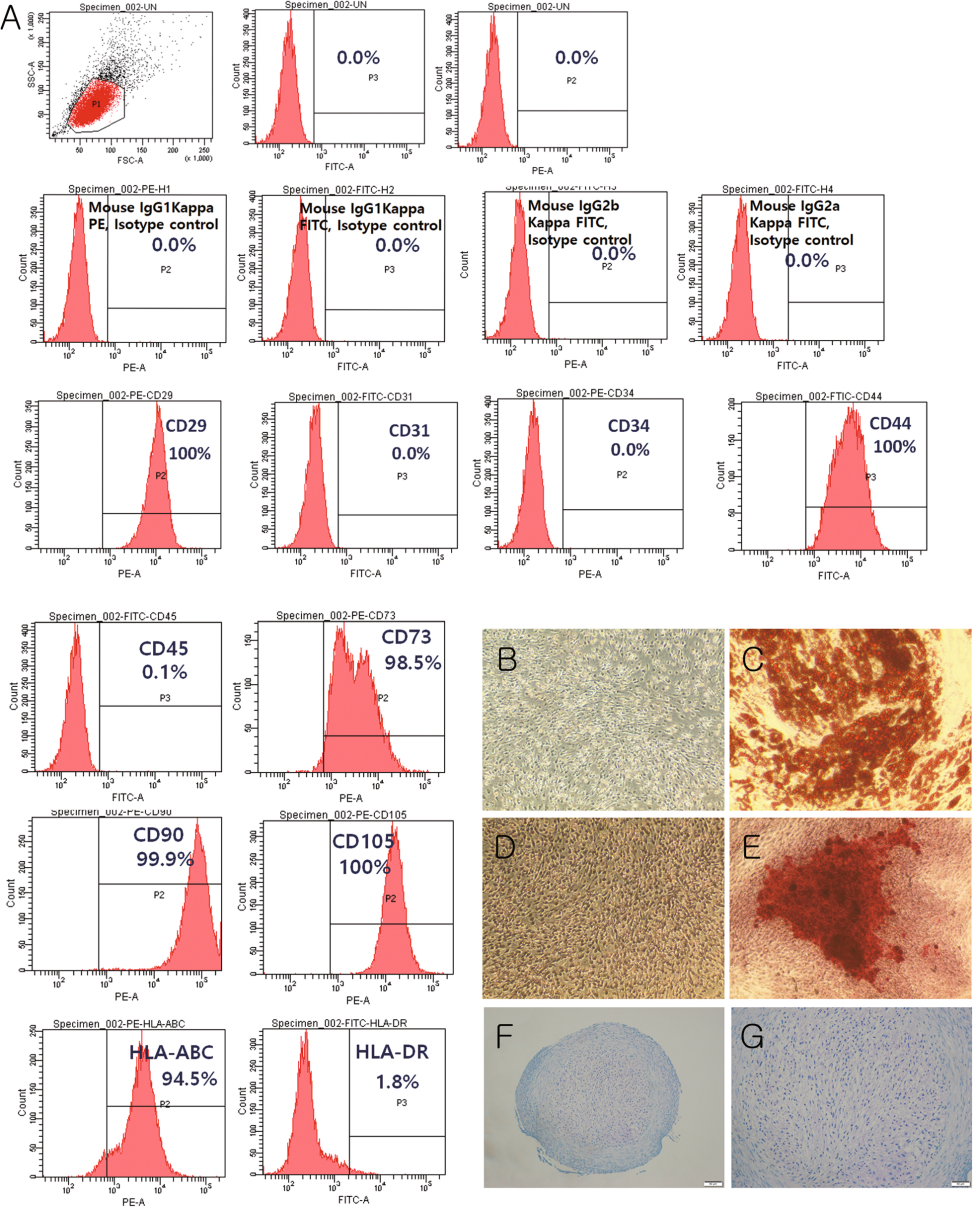
Immunophenotype and differentiation of immortalized mesenchymal stem cells (iMSCs) into adipocytes, osteocytes, and chondrocytes. **A** Immunophenotype of iMSCs **B** iMSC negative control, oil red-O stain × 100 **C** iMSCs that differentiated into adipocytes contained lipid droplets, oil red-O stain × 100 **D** iMSC negative control, Alizarin red S stain × 100 **E** iMSCs differentiated into osteocytes, Alizarin red S × 100; Scale bar = 100 μm **F** Aggregated chondrocytes derived from iMSCs, toluidine blue × 100; Scale bar = 100 μm **G** Aggregated chondrocytes derived from iMSCs, toluidine blue × 200; Scale bar = 50 μm

As a result of immunophenotyping using flow cytometry, iMSC showed positive expression of CD29, CD44, CD73, CD90, and CD105, and absence of CD31, CD34, CD45, and HLA-DR (Fig. 1A). Features indicating the differentiation of iMSCs into adipocytes, osteocytes, and chondrocytes are shown in Fig. 1B–G.

### Exosome characterization

**Fig. 2:**
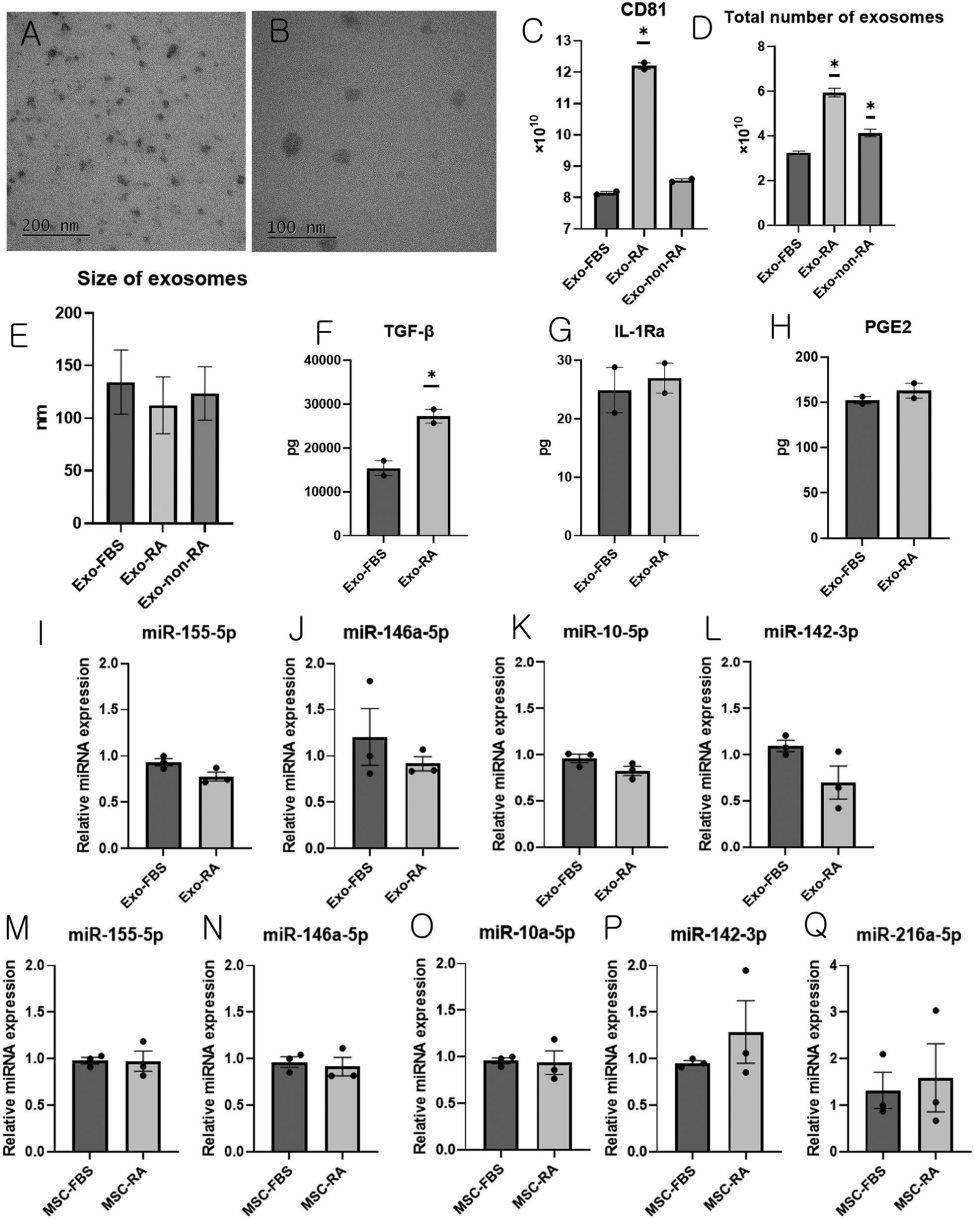
Characterization of Exosomes. **A** Transmission electron microscope analysis of freshly isolated exosomes in distilled water; Scale bar = 200 nm **B** Transmission electron microscope analysis of freshly isolated exosomes in methanol; Scale bar = 100 nm. Exosomes derived from the supernatant of iMSCs (Exo-FBS) or iMSCs primed with RA serum (Exo-RA) or non-RA serum (Exo-non-RA) **C** Total CD81 of exosome produced from 10^7^ iMSCs **D** Total number of exosomes produced from 10^7^ iMSCs as determined by NTA (n = 5) **E** Size of exosomes produced from 10^7^ MSCs as determined by NTA (n = 5) (F) Amounts of TGF-β in exosomes produced from 10^7^ iMSCs **G** Amounts of IL-1Ra in exosomes produced from 10^7^ iMSCs **H** Amounts of PGE2 in exosomes produced from 10^7^ iMSCs. (I) Relative expression of miR 155-5p in the exosomes. **J** Relative expression of miR 146-5p in exosomes. **K** Relative expression of miR 10-5p in exosomes. **L** Relative expression of miR 142-3p in exosomes. **M** Relative expression of miR 155-5p in iMSCs. **N** Relative expression of miR 146-5p in iMSCs. **O** Relative expression of miR 10-5p in iMSCs. **P** Relative expression of miR 142-3p in iMSCs. **Q** Relative expression of miR 216a-5p in iMSCs. Data (mean ± standard error of the mean) were compared using one-way analysis of variance followed by Tukey’s multiple comparison post-hoc tests.*, Significant differences (*p* < 0.05) from the Exo-FBS. iMSCs: immortalized mesenchymal stem cells, NTA: Nanoparticle Tracking Analysis, miR: micro RNA

Female cynomolgus monkeys, approximately 3 years of age and weighing 2–3 kg, were used as non-human primates to obtain disease-conditioned serum. The cynomolgus monkey RA model was established as previously described [26]. In female cynomolgus monkeys under respiratory anesthesia, serum obtained before inducing the RA model was used as non-RA serum, and serum obtained during an autopsy after establishing the RA model was used as RA-serum. Exosomes derived from the supernatant of iMSCs (Exo-FBS), iMSCs primed with RA serum (Exo-RA), or non-RA serum (Exo-non-RA) were isolated using Total Exosome Isolation Reagent. The approximate shape and size were confirmed by photographing with TEM (Fig. 2A, B), and the size distribution was confirmed using NTA.

The total amount of CD81 produced from 10^7^ iMSCs was 8.1 × 10^11^ in Exo-FBS, 12.3 × 10^11^ in Exo-RA, and 8.6 × 10^11^ in Exo-non-RA. The total amount of CD81 was significantly higher in Exo-RA than in Exo-FBS or Exo-non-RA (ANOVA & Tukey’s test, *p* < 0.001; Fig. 2C).

NTA revealed that the total number and size of exosomes produced from 10^7^ iMSCs was 3.25 × 10^10^ in Exo-FBS with an average size of 134.4 nm, 5.94 × 10^10^ in Exo-RA with an average size of 112.4 nm, and 4.13 × 10^10^ in Exo-non-RA with an average size of 123.8 nm; the number of exosomes was the highest and size was the smallest in Exo-RA (Fig. 2D, E).

### ELISA for determination of the concentration of TGF-β1, prostaglandin E2, and IL-1Ra in exosomes

Analysis of exosomes produced from 10^7^ iMSCs revealed that the amount of TGF-β1 was 15,376.4 ± 1693.2 pg in Exo-FBS and 27,242.4 ± 1564.8 pg in Exo-RA; the amount of TGF-β1 was significantly higher in Exo-RA than in Exo-FBS (Student's t-test, *p* = 0.036; Fig. 2F).

Analysis of exosomes produced from 10^7^ iMSCs revealed that the amount of IL-1Ra was 24.9 ± 3.9 pg in Exo-FBS and 27.0 ± 2.5 pg in Exo-RA (Fig. 2G). The amount of prostaglandin E2 (PGE2) levels was 152.3 ± 3.9 pg in Exo-FBS and 162.8 ± 8.3 pg in Exo-RA (Fig. 2H).

### Expression levels of miR-155-5p, miR-146a-5p, miR-10-5p, miR-142-3p, and miR-216a-5p in exosomes and iMSCs

There were no statistically significant differences in the expression levels of miR-155-5p, miR-146a-5p, miR-10-5p, and miR-142-3p between the equal concentration of RNA extracted from Exo-FBS and Exo-RA (Fig. 2I–L). There was no statistically significant difference in the expression levels of miR-155-5p, miR-146a-5p, miR-10-5p, miR-142-3p, and miR-216a-5p between equal concentrations of RNA extracted from MSC-FBS and MSC-RA (Fig. 2M–Q).

### In vitro functional test

The levels of IL-6, IL-17, IL-4, IFN-γ, IL-2, and MCP-1 were analyzed in the supernatants of ConA-stimulated splenocytes, and the levels of TNF-α, KC, IL-10, IL-1α, IL-1β, MIP-2, IL-12p70, and RANTES were analyzed in LPS-stimulated splenocytes.

**Fig. 3:**
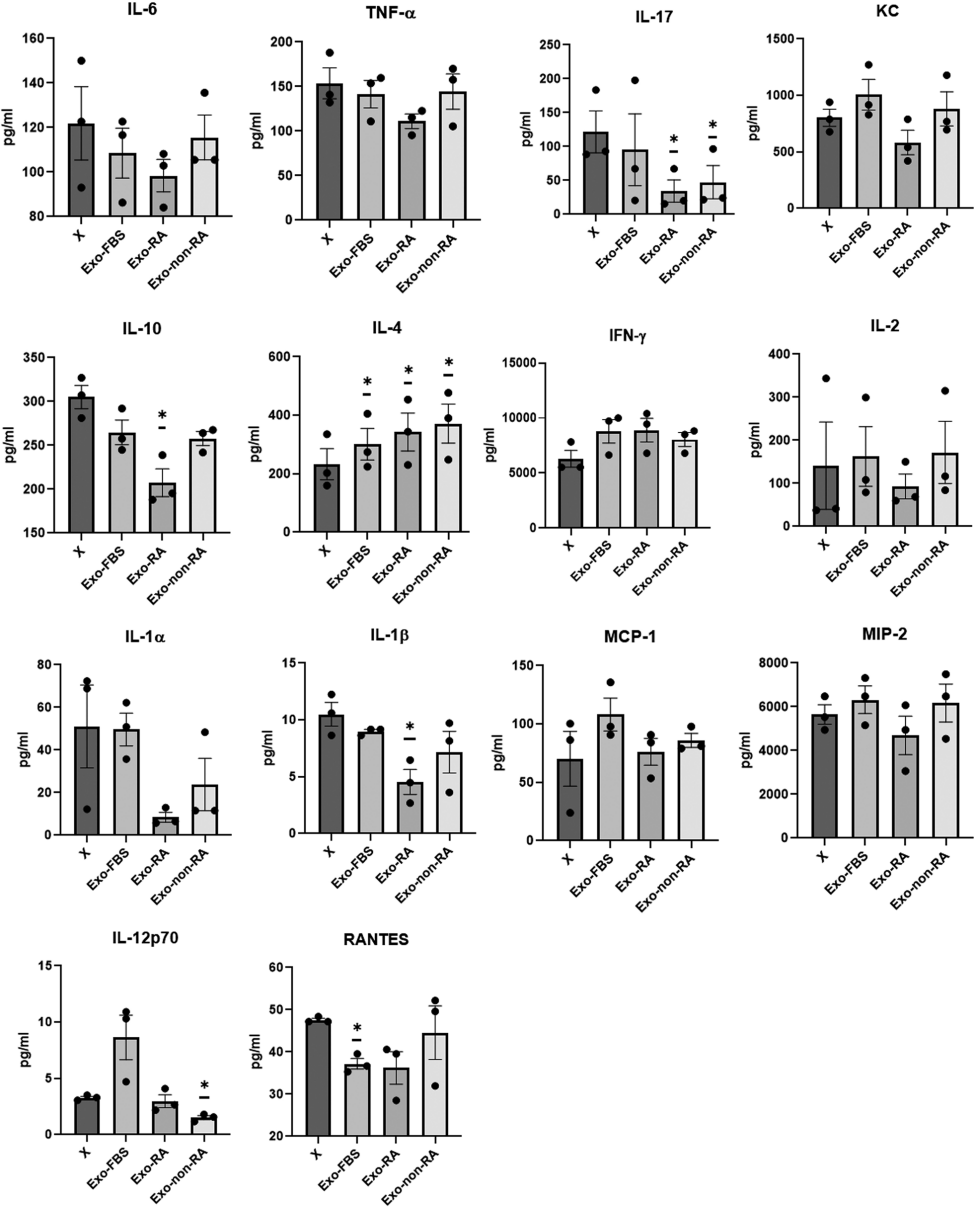
Cytokine levels in culture supernatants of splenocytes obtained from collagen-induced arthritis mouse models. The spleen cells from three mice were isolated on day 48 after CII immunization (2 × 10^5^ cells/96-well plate well) with or without ConA (5 μg/mL), or LPS (2.5 μg/mL). Each well was treated with medium (no-treatment, X), Exo-FBS, Exo-RA, or Exo-non-RA (n = 3). The exosomes and splenocytes were co-cultured at a ratio of 9 μg: 2 × 10^5^. The levels of IL-6, IL-17, IL-4, IFN-γ, IL-2, and MCP-1 were analyzed in the supernatants of ConA-stimulated splenocytes, and the levels of TNF-α, KC, IL-10, IL-1α, IL-1β, MIP-2, IL-12p70, and RANTES were analyzed in LPS-stimulated splenocytes. Data are expressed as the mean ± standard error of the mean. To compare means from two related samples, the paired *t*-test was used. *, Significant (*p* < 0.05) differences from the control (X). CII: type II collagen

The levels of RANTES were significantly lower (paired *t*-test, *p* = 0.007) and the levels of IL-4 were significantly higher in Exo-FBS-treated wells than in untreated cells (paired *t*-test, *p* = 0.001; Fig. 3).

The levels of IL-17, IL-10, and IL-1β were significantly lower (paired *t*-test, *p* = 0.026, *p* = 0.034, and* p* < 0.001, respectively) and the levels of IL-4 were significantly higher in Exo-RA-treated wells than in untreated cells (paired *t*-test, *p* = 0.034; Fig. 3).

The levels of IL-17 were significantly lower (paired *t*-test, *p* = 0.007) and the levels of IL-4 were significantly higher in Exo-non-RA-treated cells than in untreated cells (paired *t*-test, *p* = 0.04; Fig. 3).

### In vivo assay

#### Assessment of the arthritis severity

**Fig. 4:**
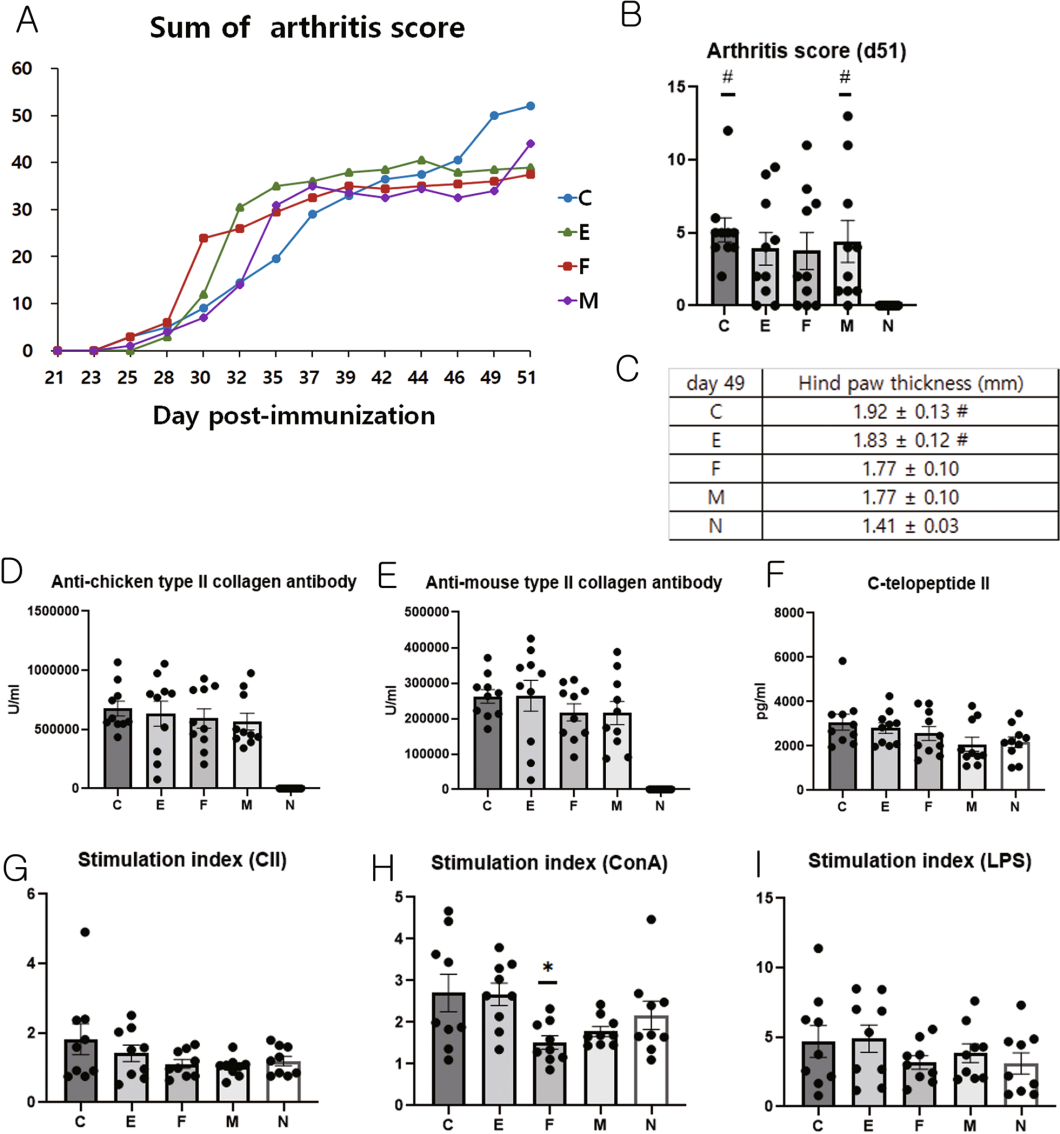
Arthritis score, concentrations of anti-type II collagen antibody and C-telopeptide, and suppression test of supernatant from spleen cells in the experimental groups. **A** Sum of arthritis score. **B** Arthritis score on day 51. **C** Hind paw thickness. **D** Concentration of anti-chicken type II collagen antibody. **E** Concentration of anti-mouse type II collagen antibody. **F** Concentration of C-telopeptide. On day 52 or 53, spleens were harvested from all mice (n = 10/group). Splenocytes from each mouse were cultured in a 96-well plate (2 × 10^5^ cells/well, final volume: 200 μL) with **G** CII, **H** ConA, or **I** LPS (100 μg/mL, 5 μg/mL, and 2.5 μg/mL respectively). Plates were incubated at 37 °C in 5% CO_2_. After 3 days, the BrdU assay was conducted. The stimulation index value (mean optical density of CII or mitogen-stimulated cultures/mean optical density of medium-only cultures) was calculated for each culture treatment. Data (mean ± standard error of the mean) were compared using one-way analysis of variance followed by Tukey’s multiple comparison post-hoc tests.*, Significant differences (*p* < 0.05) from the control (C group); #. Significant (*p* < 0.05) differences from the normal (N) group. CII: type II collagen, ConA: Concanavalin A, LPS: Lipopolysaccharide

On the last day of measurement (day 51), group C had the highest sum of arthritis score, followed by groups M, E, and F (Fig. 4A). There was no significant difference in the arthritis score of groups N, E, and F, however, group C and M had significantly higher scores than group N on the last day of measurement (arthritis score, day 51: ANOVA and Tukey’s test, *p* = 0.012, Fig. 4B).

In hind paw thickness analysis, there was no significant difference between groups N, F, and M, however, groups C and E were significantly thicker paws than group N on the last day of measurement (hind paw thickness, day 49: ANOVA and Tukey’s test, *p* = 0.008; Fig. 4C).

#### Anti-CII antibody and serum C-telopeptide II levels

Although there was no statistical significance, the mean concentration of anti-chicken CII antibody was the highest in group C, followed by groups E, F, M, and N (Fig. 4D), while the mean concentration of anti-mouse CII antibody was the highest in group E, followed by groups C, F, M, and N (Fig. 4E). The mean concentration of c-telopeptide II was the highest in group C, followed by that in groups E, F, N, and M (Fig. 4F).

#### T cell proliferation test using splenocytes from experimental animals

T cell proliferation in response to CII or LPS treatment was not significantly different among groups. However, T cell proliferation in response to the ConA stimulus was significantly suppressed in group F compared to group C (ANOVA and Tukey’s test, *p* = 0.029; Fig. 4G–I).

#### Flow-cytometric determination of T cell subset, CD138 cells, and macrophage subset in the spleen

**Fig. 5:**
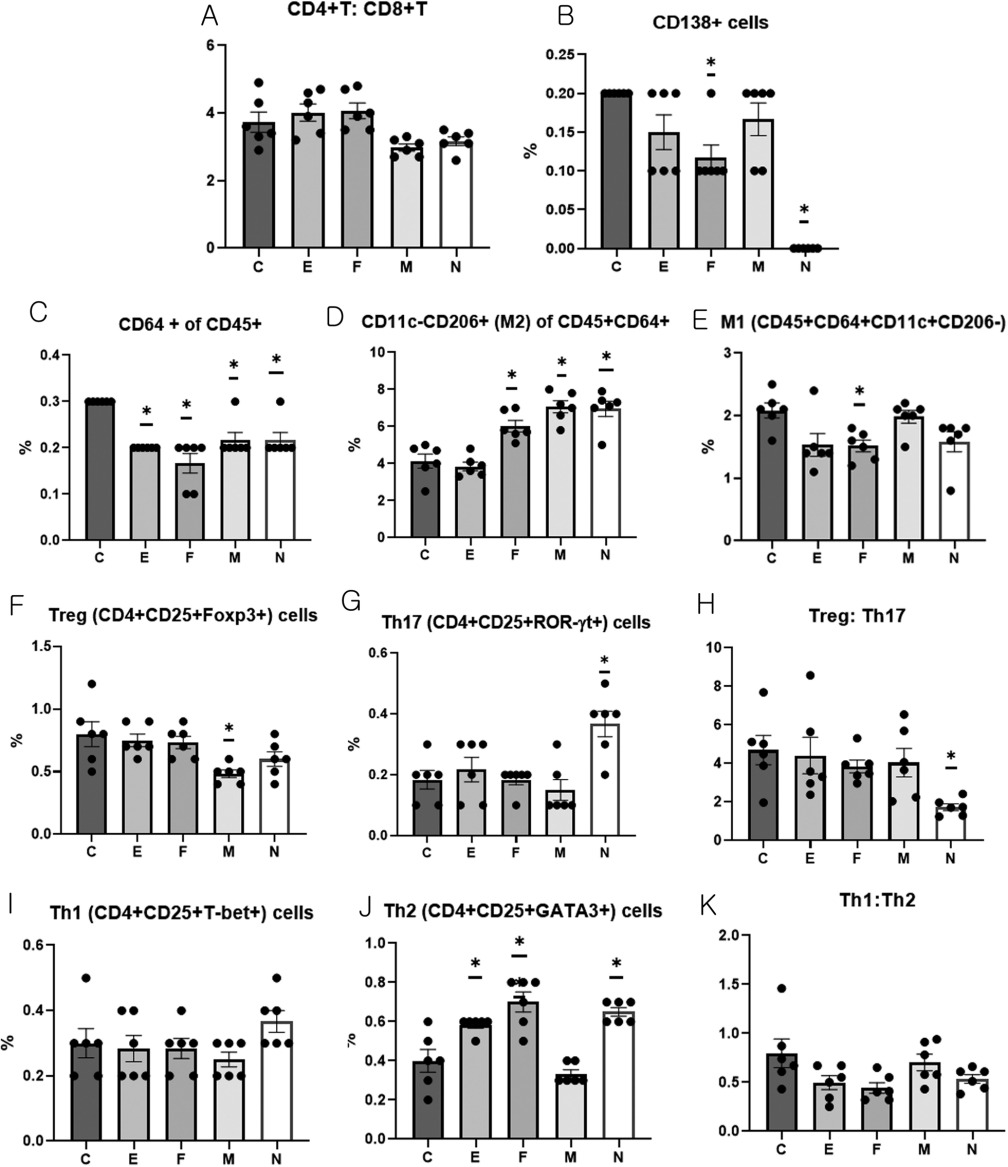
Flow-cytometric determination of T cell subsets, CD138 cells, macrophage subset, and the T helper cell subset in the spleen taken from the experimental groups. **A** CD4+T cells: CD8+T cells; **B** CD138+ cells; **C** CD64+ cells of CD45+ cells; **D** CD11c-CD206+ (M2) cells of CD45+CD64+ cells; **E** M1 (CD45+CD64+CD11c+CD206−) cells; **F** Treg (CD4+CD25+Foxp3+) cells; **G** Th17 (CD4+CD25+ROR-γt+) cells; **H** Treg:Th17 **I** Th1 (CD4+CD25+T-bet+) cells; **J** Th2 (CD4+CD25+GATA3+) cells; (K) Th1:Th2. Data obtained from experimental groups were compared using one-way analysis of variance followed by Tukey’s multiple comparison *post-hoc* tests (n = 6/group). *, Significant (*p* < 0.05) differences from the control (C group)

There was no significant difference in the CD4+:CD8+ cell ratio among the groups (Fig. 5A). CD138+ cell proportion was significantly lower in the F and N groups than in the C group (ANOVA and Tukey’s test, *p* < 0.001; Fig. 5B).

Among the CD45^+^ cells, the proportion of CD64^+^ cells was significantly lower in groups E, F, M, and N than in C group (ANOVA and Tukey’s test, *p* < 0.001; Fig. 5C). The proportion of CD11c−CD206+ macrophages (M2) among the CD45+CD64+ cells was significantly higher in the F, M, and N groups than in the C and E groups (ANOVA and Tukey’s test, *p* < 0.001; Fig. 5D). The proportion of M1 (CD45+CD64+CD11c+CD206−) macrophages was significantly lower in the F than in C group (ANOVA and Tukey’s test, *p* = 0.012; Fig. 5E).

#### Flow-cytometric analysis of the T helper cell subset in the spleen

The ratio of Tregs (CD4+CD25+Foxp3+) was significantly lower in group M than in group C (*p* = 0.008), whereas it was not different in the other groups from group C (Fig. 5F). The proportion of Th17 (CD4+CD25+ROR-γt+) cells was significantly higher in group N than in group C (ANOVA and Tukey’s test, *p* = 0.001), whereas that in the other groups did not differ from that in group C (Fig. 5G). The Treg: Th17 ratio was significantly lower in group N than in group C (ANOVA and Tukey’s test, *p* = 0.031), while it was not different in the other groups from group C (Fig. 5H).

The proportion of Th1 (CD4+CD25+T-bet+) cells did not differ among the groups (Fig. 5I), and the proportion of Th2 (CD4+CD25+GATA3+) cells was significantly higher in groups E, F, and N than in group C and M (ANOVA and Tukey’s test, *p* < 0.001; Fig. 5J). The Th1:Th2 ratio was significantly different among the groups (ANOVA, *p* = 0.041); it was the highest in group C, followed by groups M, N, E, and F (Fig. 5K).

#### Cytokine levels in sera and knee joint extracts

**Fig. 6:**
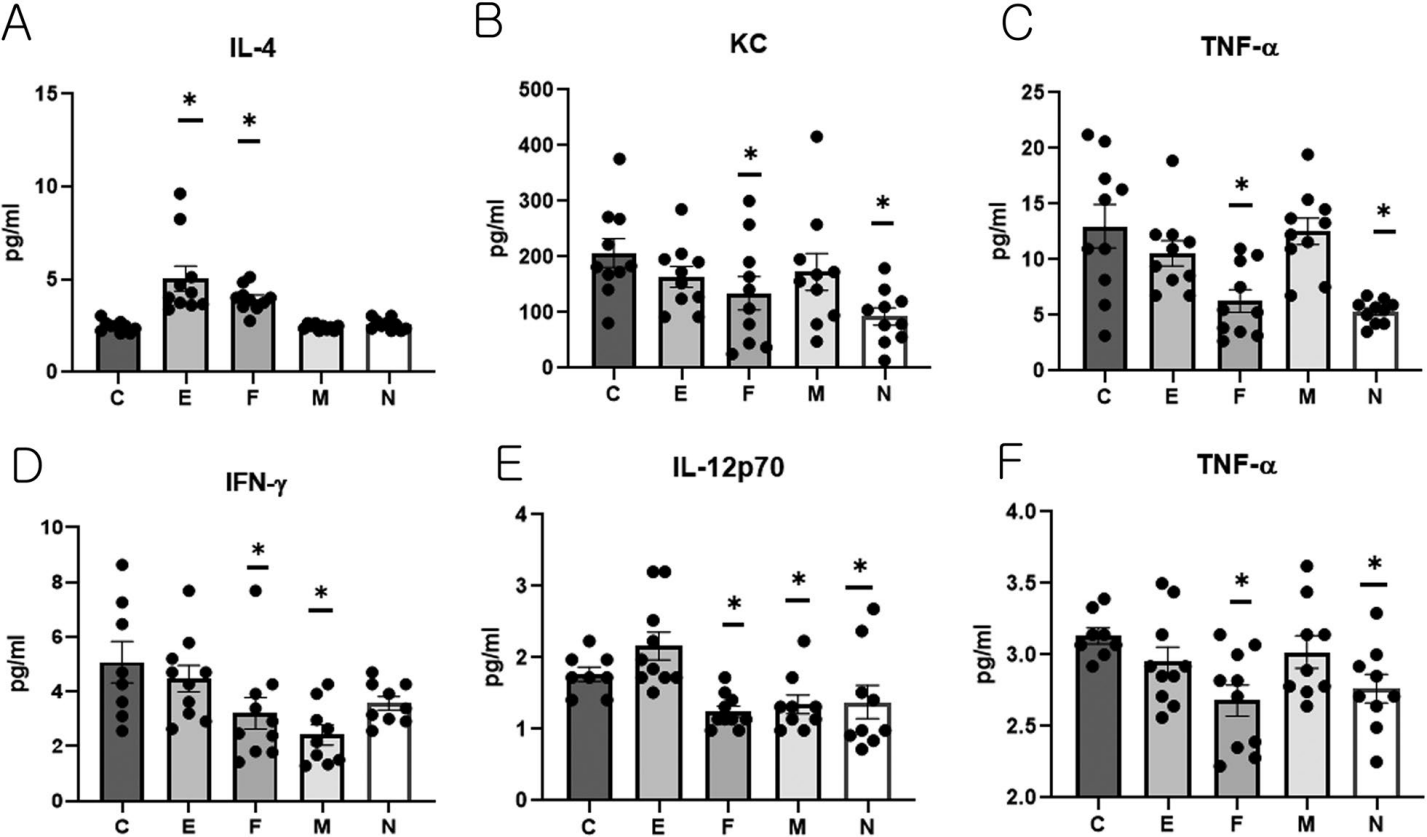
Various cytokine levels in serum and knee joint extract samples **A** Serum levels of IL-4. **B** Serum levels of KC. **C** Serum levels of TNF-α. **D** IFN-γ levels in knee joint extracts. **E** IL-12p70 levels in knee joint extracts. **F** TNF-α levels in knee joint extracts. Data obtained from experimental groups were compared using Kruskal–Wallis and Dunn’s tests (serum cytokines: n = 10/group, knee extracts: n = 8–10/group). *, Significant differences (*p* < 0.05) from the control (C group)

Serum levels of IL-4 were significantly higher in group E and F than in group C (Kruskal–Wallis test, *p* < 0.001; Dunn’s test C vs E, *p* < 0.001 and C vs. F, *p* < 0.001; Fig. 6A). Serum levels of KC and TNF-α were significantly lower in group F and N than in group C (KC: Kruskal–Wallis test, *p* = 0.019; Dunn’s test C vs F, *p* = 0.04 and C vs N, *p* = 0.001; TNF-α: Kruskal–Wallis test, *p* < 0.001; Dunn’s test C vs F, *p* = 0.003 and C vs N, *p* = 0.001; Fig. 6B and C).

The level of IFN-γ in knee joint extracts was significantly lower in groups F and M than in group C (Kruskal–Wallis test, *p* = 0.006; Dunn’s test C vs F, *p* = 0.024 and C vs M, *p* = 0.002; Fig. 6D). The level of IL-12p70 in knee joint extracts was significantly lower in group F, M, and N than in group C (Kruskal–Wallis test, *p* = 0.001; Dunn’s test C vs F, *p* = 0.016, C vs M, *p* = 0.042, and C vs N, *p* = 0.028; Fig. 6E). The level of TNF-α in knee joint extracts was significantly lower in group F and N than in group C (Kruskal–Wallis test, *p* = 0.029; Dunn’s test C vs F, *p* = 0.004 and C vs N, *p* = 0.01; Fig. 6F).

#### Histological evaluation of knee joint

**Fig. 7:**
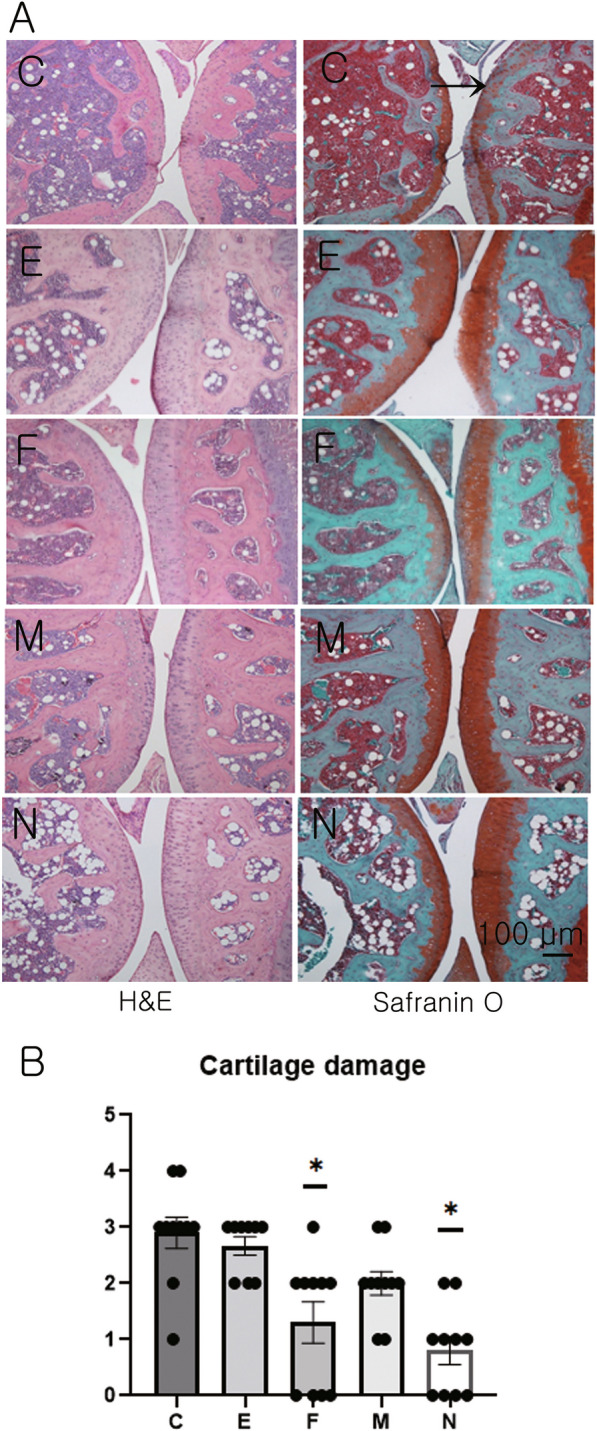
Histopathological examination of knee joint **A** Histopathology: representative knee joint sections of each group at day 52 or 53 after administration of primary immunization. Original magnification: ×100, arrowhead: severe cartilage destruction. **B** Score of cartilage damage; 0–4 (no damage to severe damage). Data obtained from experimental groups were compared using one-way analysis of variance followed by Tukey’s multiple comparison *post-hoc* tests (n = 10/group).*, Significant differences (*p* < 0.05) from the control (C group)

The knee joints of mice from both the F and N groups showed lesser destruction of articular cartilage compared to group C (Fig. 7A). The histopathological scores of the knee joints of group F and N were significantly lower than those of group C (ANOVA and Tukey’s test, *p* < 0.001; Fig. 7B).

Therefore, the administration of Exo-RA alleviated the progression of cartilage destruction in the CIA mouse model.

**Fig. 8:**
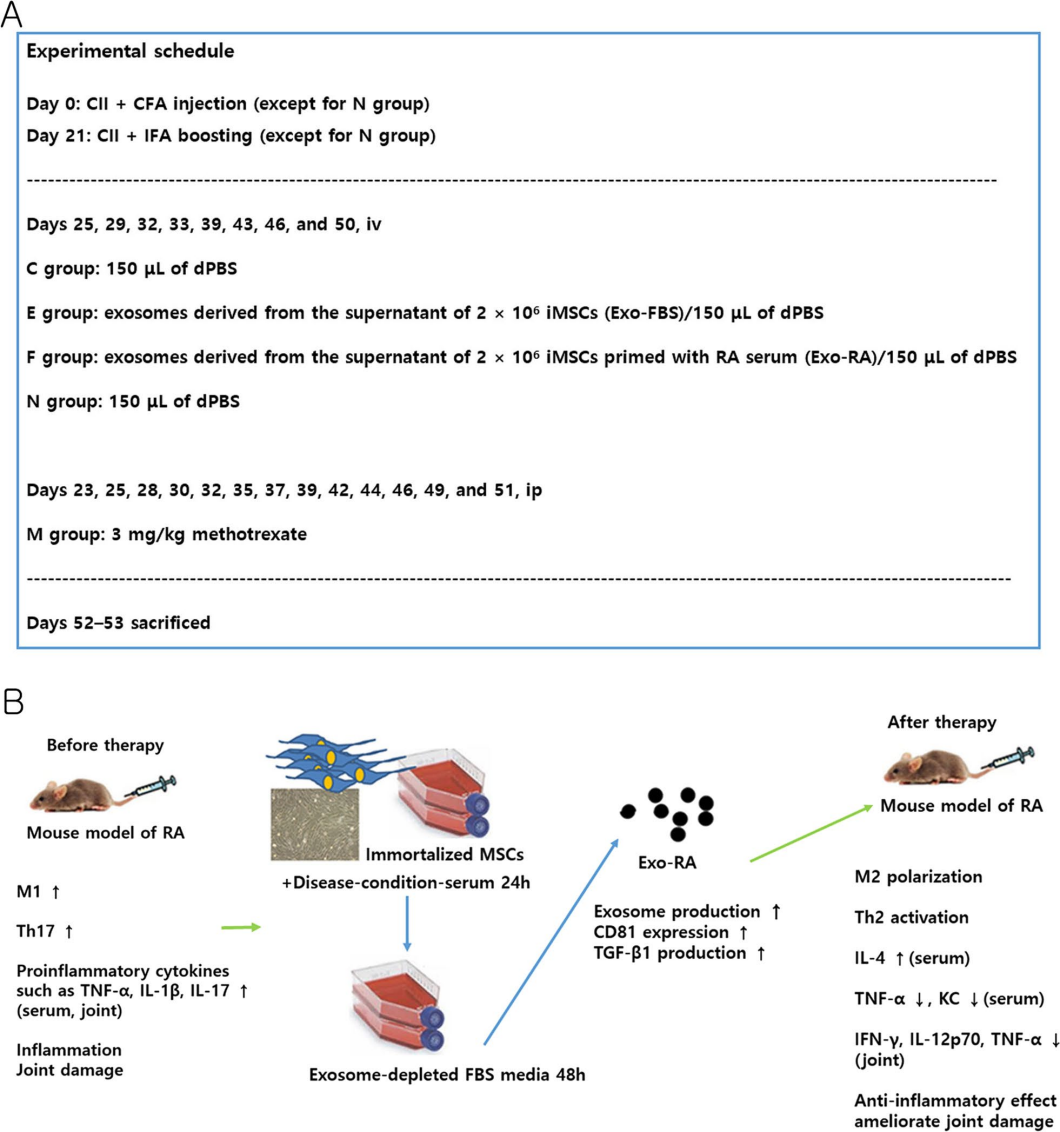
Experimental schedule and changes in cytokines after Exo-RA treatment in a mouse model of RA. **A** Experimental schedule and experimental groups according to administered material. **B** Changes in cytokines after Exo-RA treatment in a mouse model of RA. CFA, complete Freund’s adjuvant; IFA, incomplete Freund’s adjuvant. iv: intravenous injection; ip: intraperitoneal injection; FBS: fetal bovine serum; Exo-RA: exosomes derived from the supernatant of 2 × 10^6^ iMSCs primed with RA serum

Figure 8 shows a summary of the schedule of animal experiments, the production process of Exo-RA, and cytokine changes in a mouse model of RA after Exo-RA administration.

## Discussions

In RA, the dysregulation of various cytokines appears to be due to Th17/Treg and M1/M2 imbalances [27]. Th17 and M1 cells are important sources of IL-1β, IL-17, and TNF-α, which induce inflammation and joint damage in RA [28]. IL-4, an anti-inflammatory cytokine, has a significantly decreased serum concentration in patients with RA compared to that in the healthy controls and plays an immunosuppressive and anti-osteoclastogenic role in RA modulation [29].

MSCs suppress innate and adaptive immune cells; they induce an anti-inflammatory state by inhibiting T and B cells, inducing Treg, and lowering proinflammatory cytokines such as TNF-α, IL-1β, and IL-6 [30]. In addition, activated MSC induces M2 activation by increasing IL-10 [31], and MSC promotes bone regeneration by suppressing osteoclast function. MSC administration in RA improved the immune balance by lowering TNF-α, IL-1β, IL-6, and IL-17 and increasing TGF-β1 and IL-10 [32].

After administration, MSC-derived exosomes did not change in response to the host microenvironment. Hence, in the present study, we provided MSCs with a host microenvironment to MSCs prior to obtaining exosomes.

NTA and analysis of the exosome marker CD81 revealed that Exo-RA had a significantly higher number of exosomes compared to Exo-FBS, and the amounts of TGF-β1 were significantly higher in Exo-RA than those in Exo-FBS. Based on these results, we found that the exosomes obtained from MSCs pre-educated in the patient's microenvironment (disease-condition-serum) were functionally advanced.

miRNAs such as miR-155-5p, miR-146a-5p, miR-10-5p, miR-142-3p, and miR-216a-5p are known to contribute to the immunomodulatory effect of MSCs and MSC-derived extracellular vesicles in autoimmune diseases [33–35]. In this study, there was no significant difference in the expression of miR-155-5p, miR-146a-5p, miR-10-5p, and miR-142-3p in equal concentrations of RNA from Exo-FBS and Exo-RA. There was no statistically significant difference in the expression levels of miR-155-5p, miR-146a-5p, miR-10-5p, miR-142-3p, and miR-216a-5p in equal concentrations of RNA from MSC-FBS and MSC-RA. However, the number of exosomes and the total amount of CD81 were significantly higher in Exo-RA than in Exo-FBS. Although priming with disease-condition-serum did not increase immunomodulation-specific miRNA expression in the RNA from exosomes or MSCs, it significantly increased exosome production from MSCs and induced superior therapeutic effects. Several studies have reported that MSC-derived exosomes show dose-dependent effects. For example, MSC-derived exosomes enhanced functional recovery in rat traumatic brain injury in a dose-dependent manner [36].

MSCs restore the immune balance via TGF-β and PGE2 [33]. PGE2 upregulates cAMP, inhibits the proinflammatory cytokines IL-12p70 and TNF-α, and induces the anti-inflammatory cytokines IL-4 and IL-10 [37]. In addition, cAMP promotes M2 macrophage and Th2 cell differentiation, and inhibits Th1 cell production. TGF-β is the main immunoregulatory cytokine produced by MSCs. Through the TGF-β/NF-kB pathway, the production of Th17 and Th1 decreases, and the production of Treg and Th2 increases [37]. Deregulated NF-κB activation contributes to the inflammatory response in RA [38]. TGF-β antagonizes the activation of important targets of proinflammatory stimuli of NF-κB in lymphocytes and macrophages [39]. Thus, the interaction of NF-κB and TGF-β signaling pathways may be important in RA progression and treatment.

In this experiment, it was difficult to observe significant changes in Tregs or Th17 cells following exosome administration; however, significant increases in Th2 and anti-inflammatory M2 macrophages were confirmed.

When splenocytes obtained from the CIA model (RA model) were stimulated with ConA or LPS, the levels of IL-17, IL-10, and IL-1β were significantly lower and the levels of IL-4 were significantly higher in Exo-RA-treated cells than in untreated cells.

Anti-chicken type II collagen antibody and C-telopeptide levels were expected to be significantly lower in group F than in the other groups. However, the difference between the groups was not statistically significant. However, when splenocytes were stimulated with ConA, the stimulation index was significantly lower in group F than in the other groups. Therefore, the immune cell response to ConA was significantly reduced.

Serum IL-4 levels and the proportion of Th2 (CD4+CD25+GATA3+) cells were significantly increased in the Exo-FBS and Exo-RA treatment groups than in the control group. Furthermore, the proportion of anti-inflammatory M2 cells was significantly increased in the Exo-FBS and Exo-RA treatment groups than in the control group. In addition, serum levels of KC and TNF-α and the levels of IFN-γ, IL-12p70, and TNF-α in the knee joint were significantly lower in the Exo-RA treatment group than in the control group. Moreover, the histopathological scores of the knee joints were significantly lower in the Exo-RA treatment group than in the control group.

TNF-α plays an important role in the pathogenesis of RA. In patients with RA, TNF-α is primarily produced by macrophages that are activated by synovial tissue inflammation and is capable of inducing the production of other proinflammatory cytokines [40]. Accordingly, TNF-α induces the synthesis of KC, which in turn acts as a neutrophil chemoattractant [41].

Therefore, in this study, a decrease in serum KC and TNF-α levels induced a reduction in the inflammatory response and recovery in the RA model.

IL-12p70 levels are elevated in both serum and synovial fluid of patients with RA [42]. IL-12 induces the production of other proinflammatory cytokines and IL-12 levels have been reported to reflect RA disease activity [42, 43]. IL-12p70 plays a major role in inducing Th1 cells by promoting the production of IFN-γ from T cells that infiltrate the joints of patients with RA [44].

Therefore, a decrease in the serum levels of KC and TNF-α, and in the levels of IL-12p70 and TNF-α in the joints induced a reduction in the inflammatory response and recovery from RA. MSC-derived extracellular vesicles suppress the proliferation and differentiation of B cells, and macrophages transform into an anti-inflammatory M2 phenotype via the PI3K-AKT pathway [33, 45, 46]. miRNA-155-5p and miRNA-216a-5p derived from MSC-derived extracellular vesicles contribute to this mechanism [33].

Regarding the clinical symptoms related to arthritis, the control, and Exo-FBS groups had significantly thicker paws than the normal group, whereas there was no difference in paw thickness between the Exo-RA group and the normal group. Compared with the control group, the Exo-RA group showed improved arthritis score sum and average foot thickness, but the difference was not statistically significant. It is believed that a higher dose or more frequent administration of exosomes is necessary to obtain a greater inhibitory effect on clinical symptoms.

In previous studies on MSC-derived exosomes related to RA, strategies to overexpress miRNAs have been used to enhance the therapeutic effect of exosomes. Because miR-192 is known to inhibit the growth of RA fibroblast-like synoviocytes, an MSC-derived exosome overexpressing miR-192-5p was constructed [47]. Because miR-150-5p significantly reduced the expression of MMP-14 and VEGF in RA fibroblast-like synoviocytes, an MSC-derived exosome overexpressing miR-150-5p was constructed [48]. When MSC-derived exosomes overexpressing miR-150-5p or miR-192 were applied to an RA model, the treatment effect was greater than that of MSC-derived exosomes alone [47, 48]. When MSC-derived exosome overexpressing miR-192-5p was administered, a significant decrease in serum TNF-α was observed in a RA model [47].

Our results suggest that exosomes derived from MSCs primed with disease-condition-serum are highly functional and that MSC-derived exosomes or exosomes derived from MSCs primed with disease-condition-serum shift immune cells to Th2 and M2 differentiation in the RA model. Furthermore, exosomes derived from MSCs primed with disease-condition-serum may alleviate cartilage damage by lowering the concentration of proinflammatory cytokines such as TNF-α, KC, and IL-12p70.

When applied to human medicine, patient serum can be used as an iMSC priming strategy to obtain more efficient exosomes for treatment of RA.

## Conclusions

Exosomes derived from disease-condition-serum-primed iMSCs ameliorated cartilage damage in a RA model by enhancing TGF-β1 production, inducing Th2 and M2 polarization and lowering proinflammatory cytokines, TNF-α, KC, and IL-12p70 in the host. Patient-derived serum can be used as an iMSC priming strategy in the iMSC-derived exosome treatment of RA.

## Data Availability

The data used in this study are available from the corresponding author upon reasonable request.

## Abbreviations

RA: Rheumatoid arthritis
MSCs: Mesenchymal stem cells
IFN-γ: Interferon-γ
IL: Interleukin
MSC-exosomes: MSC-derived exosomes
*hTERT*: Human telomerase reverse transcriptase
iMSCs: Immortalized human adipose tissue-derived MSCs
Exo-FBS: Exosomes derived from the supernatant of iMSCs
Exo-RA: iMSCs primed with RA serum
Exo-non-RA: iMSCs primed with non-RA serum
BCA: Bicinchoninic acid
NTA: Nanoparticle tracking analysis
TEM: Transmission electron microscopy
TGF: Transforming growth factor
miR: MicroRNA
IACUC: Institutional Animal Care and Use Committee
CII: Chicken type II collagen
CIA: Collagen-induced arthritis
ConA: Concanavalin A
LPS: Lipopolysaccharide
C-telopeptide II: C-telopeptide of type II collagen
TNF-α: Tumor necrosis factor-α
KC: Keratinocyte chemoattractant
MCP-1: Monocyte chemoattractant protein 1
MIP-2: Macrophage inflammatory protein 2
RANTES: Regulated upon activation normal T cell expressed and presumably secrete
MTX: Methotrexate
M1: M1 macrophages
M2: M2 macrophages
Th: T helper
Treg: T regulatory cells
SEM: Standard error of the mean
ANOVA: One-way analysis of variance
cAMP: Cyclic AMP
PI3K: Phosphatidylinositol-3 kinase
NF-kB: Nuclear factor kappa light chain enhancer of activated B cells
